# Unifying a fragmented effort: a qualitative framework for improving international surgical teaching collaborations

**DOI:** 10.1186/s12992-017-0296-7

**Published:** 2017-09-07

**Authors:** Parisa Nicole Fallah, Mark Bernstein

**Affiliations:** 1 Harvard Medical School, 25 Shattuck Street, Boston, MA 02115 USA; 2 Division of Neurosurgery, Department of Surgery, University of Toronto, Toronto, ON Canada; 3 Division of Neurosurgery, Toronto Western Hospital, University Health Network, Toronto, ON Canada

**Keywords:** Global health, Global surgery, Surgery, International teaching collaborations, Low- and middle-income countries, Sustainability, Organization, Systematization, Unification, Quality improvement

## Abstract

**Background:**

Access to adequate surgical care is limited globally, particularly in low- and middle-income countries (LMICs). To address this issue, surgeons are becoming increasingly involved in international surgical teaching collaborations (ISTCs), which include educational partnerships between surgical teams in high-income countries and those in LMICs. The purpose of this study is to determine a framework for unifying, systematizing, and improving the quality of ISTCs so that they can better address the global surgical need.

**Methods:**

A convenience sample of 68 surgeons, anesthesiologists, physicians, residents, nurses, academics, and administrators from the U.S., Canada, and Norway was used for the study. Participants all had some involvement in ISTCs and came from multiple specialties and institutions. Qualitative methodology was used, and participants were interviewed using a pre-determined set of open-ended questions. Data was gathered over two months either in-person, over the phone, or on Skype. Data was evaluated using thematic content analysis.

**Results:**

To organize and systematize ISTCs, participants reported a need for a centralized/systematized process with designated leaders, a universal data bank of current efforts/progress, communication amongst involved parties, full-time administrative staff, dedicated funds, a scholarly approach, increased use of technology, and more research on needs and outcomes.

**Conclusion:**

By taking steps towards unifying and systematizing ISTCs, the quality of ISTCs can be improved. This could lead to an advancement in efforts to increase access to surgical care worldwide.

**Electronic supplementary material:**

The online version of this article (10.1186/s12992-017-0296-7) contains supplementary material, which is available to authorized users.

## Background

Approximately 5 billion people lack adequate access to surgical care [1]. This issue disproportionately affects those in low- and middle-income countries (LMICs), such that the poorest 30% of the world receives only about 3.5% of surgical procedures [2]. This has led to the loss of 77.2 million disability-adjusted life years (DALYs) and 16.9 million lives each year, with profound social and economic implications. Unfortunately, those who become disabled due to lack of surgical care can no longer participate effectively in the workforce. Subsequently, individual families fall deeper into poverty, and as this issue affects entire populations, LMICs face difficulties in advancing economically. If the lack of access to surgical care worldwide is not addressed, the global economy is estimated to lose 20.7 trillion dollars between 2015 and 2030, with 12.3 trillion dollars being lost from LMICs alone [3]. This would have a significant negative impact on the development of LMICs and would perpetuate global economic inequities that have persisted for many years.

The global burden of surgical disease is being addressed in several ways, outlined specifically in Chapter 13 “Specialized Surgical Platforms” of “Essential Surgery: Disease Control Priorities” [4]. One way is through humanitarian work on the part of surgeons from high-income countries (HICs) going to LMICs for shorter missions and performing surgery on patients [4–6]. For example, Dell Children’s Global Surgical Outreach goes to Guatemala several times a year and does a large volume of pediatric surgery cases [7]. This work can also involve major organizations like Operation Smile, dedicated to providing year-round care for specific surgical needs via scheduled rotations of surgical teams [8].

Although there are direct benefits to providing surgical care in LMICs, there may also be unintended consequences in the long-term [4, 6, 9]. Not all patients are able to receive operations during surgical missions, and those that do may not receive follow-up care, because the necessary resources are not always in place to provide it [10]. Local surgeons may feel demotivated when local patients are treated as a part of surgical care missions that they are not involved in [11]. Some groups in HICs also provide surgical equipment/resources for LMICs; however, it is generally difficult to keep that equipment running, often due to the lack of technicians and parts. These interventions do have an important place within global surgery efforts and provide some immediate relief for the burden of surgical disease, but they may not have long-term benefits [4]. Currently, the field of global surgery has put an increasing focus on building capacity and surgical infrastructure with long-term goals for improving access to surgical care worldwide.

An education-based solution may be more sustainable for addressing the global burden of surgical conditions [12]. Currently, 2.2 million more surgeons, anesthesiologists, and OB/GYNs are needed to address the global need for surgery [3]. Without the proper surgical workforce, it may be difficult to scale up surgical care to meet the World Health Organization's and Lancet Commision on Global Surgery's goals for 2030 [3, 13]. In addition to providing clinical care in LMICs, efforts are becoming increasingly focused on teaching surgery and helping set up surgical residency programs through effective partnerships [14, 15]. There are many ways in which this is being done. Some have created online education platforms for surgical residents in LMICs, while others have brought surgical residents from LMICs to HICs for specialized training. International surgical teaching collaborations (ISTCs) focus on building surgical education infrastructure in LMICs to address the needs for clinical care, training, and research – the “tripartite needs” of global health – and often times also utilize some of the above-mentioned modalities to achieve those goals [16].

ISTCs can increase surgical capacity in LMICs to alleviate some of the social and economic repercussions of lack of access to surgical care [14]. ISTCs involve working with local surgical providers in LMICs and adapting the education to local needs, which can help reduce brain drain [16]. The goal is for sustainable and systematic collaborative relationships to be formed to help increase the surgical capacity of LMICs [16, 17].

Currently, ISTCs do not have a standard operational process. On a small scale, a surgical team from a HIC may partner with a team from a LMIC to teach surgical procedures that they want to learn through a short training course – for example, Dr. Mark Bernstein teaches the awake craniotomy in many low-resource settings around the world [18]. This helps address very specific needs in a local setting, often the most pressing issues, and can be significantly valuable to patients in that locality. On a larger scale, there are a variety of models for ISTCs. The Foundation of International Education in Neurological Surgery (FIENS) is an organization dedicated to developing neurosurgical education worldwide [19, 20]. The Toronto Addis Ababa Academic Collaboration (TAAAC) is a full institutional collaboration that has led to the development of education programs in many different medical and surgical specialties at Addis Ababa University in Ethiopia through partnership with the University of Toronto [21]. This larger scale model focuses on building the surgical capacity of major academic institutions in LMICs. However, by also focusing on training future trainers, ISTCs can build the capacity of those institutions to reach out to underserved rural areas of their countries [21]. These larger collaborations often involve not only local stakeholders, such as surgeons, anesthesiologists, nurses, and hospital staff, but national stakeholders as well, including the Minister of Health, Minister of Finance, and others who can provide a more comprehensive perspective on the progress of these efforts.

Health partnerships have been a part of global health for many years, but have only recently become a significant part of global surgery efforts. Since ISTCs are becoming more prevalent, there are challenges that need to be resolved. Global surgery efforts have been fragmented, because the tendency towards horizontal interventions focused on building health systems and infrastructure, as opposed to vertical interventions focused on specific diseases, has increased more in recent years [22]. Thus, global surgery efforts are often individually initiated, planned, and carried out. There is minimal organization, systematization, or communication between groups. This is increasingly becoming an issue as efforts expand and overlap. Organizations such as the Lancet Commission on Global Surgery (LCoGS), Global Partners in Anesthesia and Surgery (GPAS), Program for Global Surgery and Social Change (PGSSC), and others are working towards making surgery a global health priority and towards increasing recognition of the value of educational collaborations in achieving more equity in the distribution of surgical care worldwide [23]. The LCoGS has added considerable amounts of research to the field and published a landmark paper in 2015 that led to a surge of interest and involvement in global surgery [3]. PGSSC similarly has more recently added research to the field but has also worked with LMICs to create and implement national surgical, obstetric, and anesthesia strategic plans that were suggested by the Lancet Commission’s work. GPAS is one of the earlier global surgery organizations that is geared towards capacity-building, research, and harmonization of global surgery efforts. Additionally, professional bodies such as the College of Surgeons of East, Central, and Southern Africa (COSECSA), the West African College of Surgeons (WACS), the Royal College of Surgeons (RCS), and the American College of Surgeons (ACS), are increasingly pushing the agenda of global surgery in the academic arena.

Priority needs to be given to fostering a unified approach to ISTCs by working off of the currently fragmented system [24]. In this paper, we seek to examine the elements of a framework for unifying, systematizing, and improving the quality of international surgical teaching collaborations from the perspective of those in HICs. Our hope is for a similar study to be carried out in LMICs such that the combined results can inform the next steps in sustainable global surgery efforts.

## Methods

### Study participants and sample size

Study participants included surgeons from multiple specialties (general, orthopedics, OB/Gyn, neurosurgery, cardiothoracic, oncology, plastics), anesthesiologists, other physicians (family medicine, emergency medicine, palliative care, radiology, psychiatry), residents, nurses, academics, and administrators from 20 academic medical institutions in the U.S., Canada, and Norway.

Study participants were known by the senior author and were recruited via convenience sampling. 124 people were emailed to participate in the study, and the final sample size was 68. 92 participants responded to the email, 23 did not respond, and 9 were unable to find time to schedule the interview. 68 of those interviewed had involvement in international medical or surgical teaching collaborations in some capacity, and the other 24 participants’ data was not included in this analysis. Of the participants, 62 were healthcare providers involved in international medical or surgical teaching collaborations (40 surgeons, 5 anesthesiologists, 11 additional physicians, 4 surgery residents, 2 nurses). The remaining 6 participants included academics and administrators. “Involvement” in international medical and surgical teaching collaborations included participants who had a current continued and sustained commitment.

### Study aim, design, and setting

From June 1–August 1, 2015 in Toronto, Canada, P.F. conducted 68 semi-structured interviews in-person, over the phone, or over Skype. In-person interviews took place in the participants’ academic offices within their hospitals, and phone/Skype interviews were conducted in an office space within Toronto Western Hospital. Toronto was chosen due to the location of the senior author; however, participants were not limited to Toronto as phone calls and Skype conversations allowed for a broader reach. 68 interviews were sufficient to reach data saturation, as themes began to repeat amongst the different interviews.

Questions were based on an interview guide developed by P.F. and M.B. and were intended to be open-ended to gain more in-depth responses. Demographic data collected included age, gender, specialty, and number of years in practice. Interviews were approximately 40 min long on average and were audio-recorded. No repeat interviews were carried out. Interviews were transcribed by P.F. from August 2015 to November 2015. Transcripts were not returned to participants for comment or correction. The complete list of interview questions is attached as Additional file 1.

### Data analysis

Transcripts of the audio files were analyzed by P.F. using content analysis [25, 26]. This included finding, examining, and recording patterns in the data. Themes were determined inductively after coding the data. Data integrity was verified by M.B., who has substantial experience in qualitative research methodology. Software was not used for the data organization and coding. Study participants were not asked to provide feedback on the findings.

### Ethics approval and consent to participate

Each participant gave either written or oral informed consent to participate in the study. All recordings and transcripts were de-identified and stored confidentially, and the recordings and transcripts were kept in a secure location. The study was approved by both the Research Ethics Board at the University Health Network in Toronto, Ontario, Canada (reference number 15–9030-AE) and by the Institutional Review Board at the University of Texas at Austin, Austin, Texas, USA (reference number 2015–05-0040).

### Study limitations

A convenience sample was used for the study, so there was slightly larger representation from one locality. Additionally, due to qualitative methodology, the results may not be generalizable. The large sample size was intended to help address these limitations. In general, the findings from qualitative research rely on the skills of the researcher, which may lead to some bias in the results. There is also more difficulty in assessing the rigor of the study since quantitative measurements were not used. It is also implausible to visually characterize the qualitative findings. However, qualitative research provides a more in-depth analysis of the practical issues within global surgery that quantitative measurements cannot characterize.

Another limitation is that LMICs were not included in the study. The purpose of the study was to characterize the issues causing fragmentation in global surgery efforts from the perspective of those in HICs, which is why only participants from HICs were interviewed. The perspective of those in LMICs is vital to this discussion; however, separately characterizing issues experienced by LMICs can allow those issues to be addressed specifically. A similar study should be done in LMICs to assess needs for unifying, systematizing, and improving global surgery efforts, and the authors are presently aiding colleagues in LMICs with this effort.

## Results

Six themes emerged in regards to unifying, systematizing, and improving the quality of ISTCs: consolidation, communication and collaboration, a system of support, a scholarly approach, increased use of technology, and concerns/hesitations. Some themes included subthemes, as presented below.

### Consolidation

Consolidation refers to the need for having information in a centralized location that is accessible to all of those involved in ISTCs.

#### Sub-theme: *There is a need for a centralized global surgery platform with designated leaders*

Participants reported that there are no central global surgery offices at their institutions, so the efforts are too fragmented and each hospital has its own projects. Participants felt that structure was necessary to create priorities and that it was the institution’s responsibility to implement structure. They expressed that there should be a few flagship projects from each institution with more coordination and focus.

*“I think if we appointed a head of global health and gave that person some resources and a mandate, then you have a structured global health initiative.”* –OB/Gyn, Canada

#### Sub-theme: *There is a need for a universal data bank of global surgery activities*

Participants mentioned a need for a centralized website that lists those involved in ISTCs and capacity-building efforts by specialty and country. The website would also need to include past, present, and future ISTCs so that surgical healthcare providers could easily connect rather than having to look through multiple sources.

*“In this day and age, we need a website that really takes into account every volunteer opportunity in the world and every volunteer in the world that is planning to go.”* –Neurosurgeon, USA

Participants felt this could facilitate teamwork and learning, and that it could avoid duplication. A website could also help match the need for specific surgical specialties in LMICs with the available surgical healthcare providers in HICs who wish to help address that particular need.

### Communication and collaboration

Communication and collaboration refers to the need for those involved in ISTCs to disseminate their findings to the public, including to any groups of significance to ISTC-related efforts.

#### Sub-theme: *There is a need for ISTCs and global surgery efforts to be made public*

Participants reported that meetings to reflect on the progress of ISTCs would be beneficial. Specifically, participants felt that they should more regularly discuss the goals and social values of ISTCs, as well as the effectiveness of current approaches and strategies for improvement.

*“Finding out what made [particular ISTCs] work and rolling out more of those [positive elements] would be much more efficient than everybody trying to figure it out by themselves.”* –Neurosurgeon, USA

#### Sub-theme: *There is a need for improved collaboration between involved individuals, communities, hospitals/institutions, governments, and donors*

Participants reported a need for all parties involved in ISTCs to communicate and collaborate more effectively. Participants emphasized the particular importance of having government support for ISTCs, as most governments have control over healthcare in LMICs.

*“I think the overall answer is that it requires different parties coming together. It’s a lot of collaboration, a lot of interconnectivity, a lot of liaising at community levels all the way up to governments and donors.”* –Global Surgery Administrator, Canada

Participants also mentioned the importance of collaboration in reducing redundancy of efforts. They shared that often times, they do not know about colleagues’ involvement in ISTCs within their own departments, making it difficult to support others’ efforts.

### A system of support

A system of support refers to the need for funds and the need for administrative staff that can dedicate time to organizing ISTCs.

#### Sub-theme: *There is a need for administrative staff dedicated to global surgery*

Participants mentioned a need for administrative staff to help sustain ISTCs. They reported that administrators could plan and organize trips, reach out to donors, and build departmental global surgery plans. Participants also reported that administrative staff could help form ISTCs by doing initial outreach and creating partnerships with institutions abroad.

*“If we can bring in some administrative people or some kind of political involvement, and also have some kind of knowledge on how to create these global partnerships, I think it’ll be so much more helpful.”* –Global Surgery Administrator, Canada

#### Sub-theme: *There is a need for organized and dedicated funds for global surgery*

Participants reported the need for more money to support global surgery and that it is difficult to secure the necessary funds due to the long-term nature of ISTCs.

*“We do get into a trap of always having to come up with dollars, so that certainly is a difficult thing. I would like to see more money in the department of surgery to support global surgery, as it is becoming an extremely important topic around the world.”* –Pediatric Neurosurgeon, Canada

### A scholarly approach

A scholarly approach refers to the need for an academic orientation towards global surgery.

#### Sub-theme: *There is a need for global surgery to be recognized as a professional, academic, and scholarly field*

Participants felt that global surgery needs to be professionalized. They pointed out that there should be minimum standards for ISTCs in order to avoid possible negative ramifications for LMICs. Participants also mentioned the need for global surgery to be recognized as an academic field so that it could encourage more research and formal study.

*“I think [ISTCs] are great as long as there’s an academic vent to what you’re doing, and I think there should be, because anything new that we do is open to and potentially should be studied in some formal way.”* –Neurosurgeon, Canada

#### Sub-theme: *There is a need for an interdisciplinary approach to ISTCs*

Participants reported that many different academic fields involved in global surgery work are not engaged collaboratively. They felt that ISTCs could be significantly enhanced by interdisciplinary involvement. Participants mentioned the need to think broadly about different disciplines involved in surgical work and to apply those same components to ISTCs.

*“Part of it is to engage communities that are in siloes that don’t often engage in these things together. The other part of it is to start coming up with a shared language, perspective, and attitude towards international engagement.”* –Medical Education Researcher, Canada

#### Sub-theme: *There is a need for ISTCs to be true partnerships with a bidirectional exchange of knowledge*

Participants mentioned the need for respect when engaging in ISTCs. They emphasized the importance of recognizing that those in LMICs know more about what can be accomplished within their own environment than visitors do. Participants pointed out that surgical healthcare providers in LMICs can perform some surgical procedures more effectively than those in HICs and that the value of their expertise should be acknowledged. Participants said they could learn how to use fewer resources for surgical procedures from their colleagues in LMICs.

*“It’s very helpful for clinicians [from HICs] to see what can be done with fewer resources, which is one of the things they can learn, since [surgeons from LMICs] have learned to be more efficient in certain things that they do.”* –Psychiatrist, Canada

#### Sub-theme: *There is a significant need for more research related to ISTCs*

Participants reported a need for research that assessed the surgical needs of specific hospitals and areas in LMICs. They wanted to know what resources those in LMICs have and whether those resources are amenable to building surgical care infrastructure. They also wanted to know what the surgical healthcare providers in these specific sites want to learn. They also mentioned the need for education research that could address how to most effectively teach surgery in low-resource settings.

Participants pointed out a need for outcomes research. They said that they need to know if ISTCs are actually improving surgical capacity in LMICs and that measuring effectiveness could help identify the best methods for conducting ISTCs. Longitudinal outcomes research also ensures that time and resources are being used efficiently. Participants mentioned the need for developing a system of metrics to consistently measure the impact of ISTCs and other capacity-building efforts.

### Use of technology

Use of technology refers to the need for incorporation of technology into ISTCs in order to facilitate collaboration for sustained periods of time over long-distance when consistent on-the-ground support is not possible.

Participants expressed that using technology allows inclusion of more surgical healthcare providers in ISTCs, because it provides an alternative to traveling and teaching. Additionally, participants mentioned that online technology and educational materials help standardize surgical education by making information readily available and more equally accessible to all. Participants reported using technology that ranged from online teaching modules to cameras in operating rooms to robotic surgery. They also pointed out that certain basic skills can be taught and certain cases can be discussed using video interfacing.

*“One of the great things about technology is that it offers every good surgeon in the world [a chance] to contribute, if they can, let’s say, record their surgery… I think technology plays a great role in the sense that it can empower people in any part of the world to be able to level the playing field, and it can allow them to be a valuable contributor.”* –Ophthalmologist, USA

Participants did point out that although technology is important, it cannot replace having a physical presence in LMICs. They felt that in-person collaboration is necessary, but that technology has the potential to maintain ISTCs over distance and time.

### Concerns

Participants raised concerns that organizing ISTCs could lead to over-regulation and bureaucracy. They mentioned potential increased costs, paperwork, and other barriers, which could lead to a decreased quantity of ISTCs.

*“But I would worry that in organizing [ISTCs] more, you’re also subjecting these efforts to regulation that just makes it harder to happen, increases the cost, and increases the time to jump through administrative procedures.”*  –Global Surgery Administrator, USA

Participants pointed out that sometimes surgical healthcare providers take an individualistic approach to ISTCs that may make it difficult to consolidate efforts. They expressed that surgical healthcare providers are driven people and want to be involved in ISTCs on their own without needing to get approval from others. An unfortunate side effect of this is that those involved in ISTCs do not disclose with each other, as they should, what they have done and what they know regarding global surgery work.

*“I think the thing is as surgeons and physicians in general, we are driven people, so we think whatever we decide to do is the best way to do it, and whatever other people are doing is a waste of resources.”* –Neurosurgery Resident, Canada

Participants also had questions about whether ISTCs should have a decentralized, technique-based focus in which one procedure is taught at multiple sites, or a centralized, site-based focus in which the comprehensive surgical needs of a site are addressed. For a decentralized approach, participants mentioned that it could allow for diversity and smaller, more manageable tasks. For a centralized approach, participants felt that it could streamline ISTCs and allow for all of those involved to take on a more organized integrated approach.

## Discussion

**Fig. 1:**
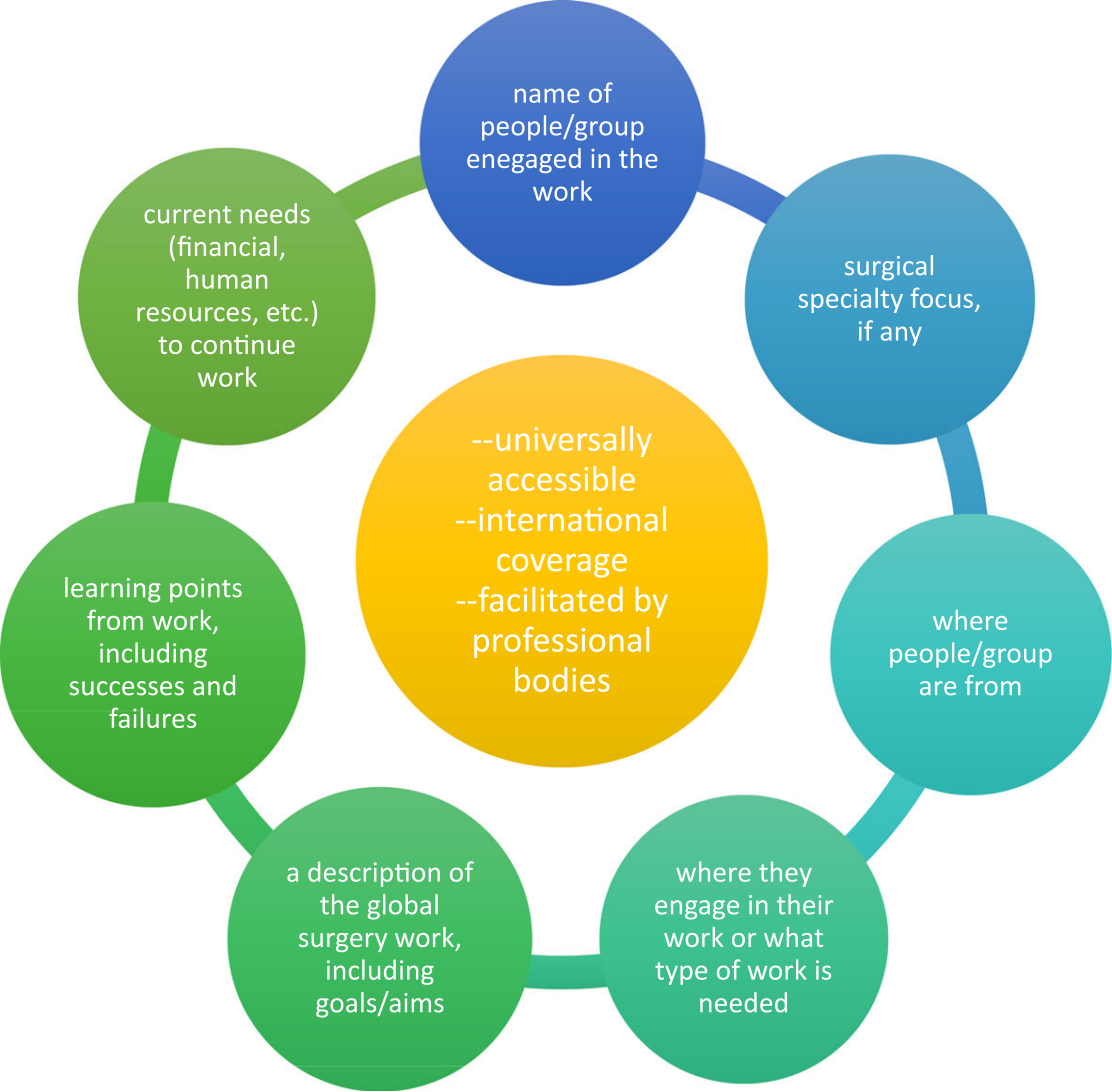
Centralized Website for Consolidating Information on ISTCs and Sustainable Global Surgery Efforts. A website for ISTCs and sustainable global surgery efforts should be universally accessible, have international coverage, and potentially be facilitated by professional bodies. The website could include names and identifying information of those involved, surgical specialty focus, where the group is from, where they are engaging in work (or what type of work is needed), a description of the global surgery work, learning points to share with others, and the current needs for sustaining the work. Other information could be included as well. This website could allow relevant stakeholders to look at current efforts, contact those involved and learn more, engage in new work based on needs, and/or initiate collaborations based on similarities of surgical specialty, regions, etc.

**Fig. 2:**
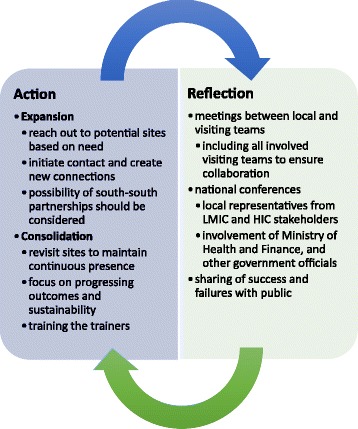
Expansion and Consolidation of ISTCs with Consistent Action and Reflection. This figure integrates the framework of action and reflection with that of expansion and consolidation. Action includes the expansion phase, which leads to more ISTCs, and the consolidation phase, which strengthens partnerships and focuses on long-term outcomes. Reflection includes meetings and conferences from the local to the national levels in order to assess the successes and failures of interventions

**Fig. 3:**
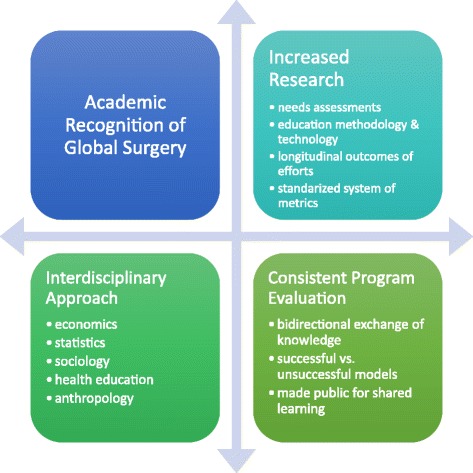
A Scholarly Approach to Global Surgery Efforts. A scholarly approach to global surgery work can further formalize efforts. Integral to this is increasing academic recognition of global surgery, increasing research on needs and outcomes, an interdisciplinary approach that engages multiple relevant fields (all of which are not listed), and consistent program evaluation

**Fig. 4:**
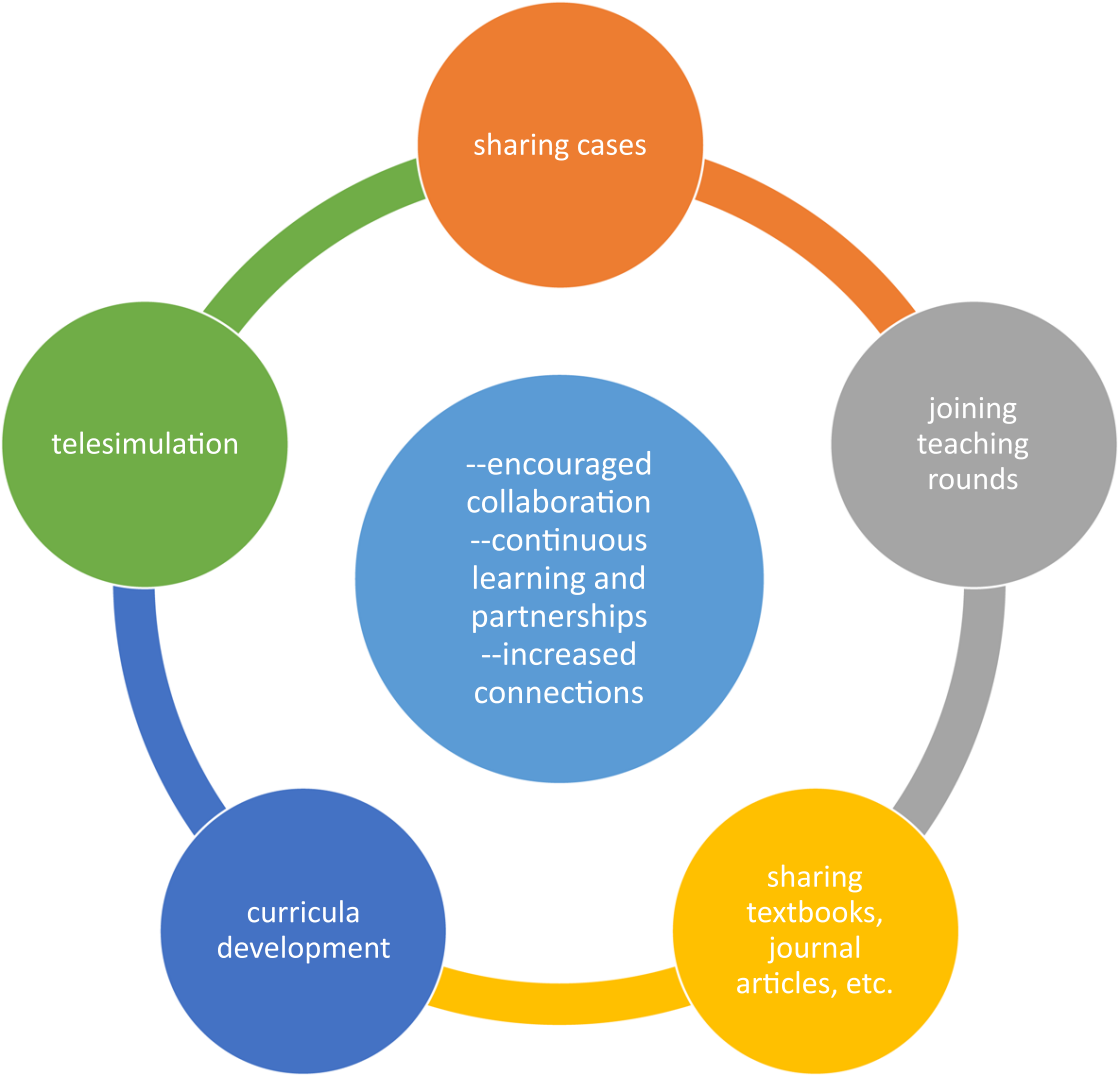
The Role of Technology in Facilitating Continued Long-Distance Partnerships. Sharing cases, having joint teaching rounds, sharing textbooks/journal articles, developing curricula, and engaging in telesimulation are all ways in which technology can be used to further advance ISTCs and global surgery efforts, although there are many other possibilities. Use of technology can encourage collaboration, lead to continuous learning and partnerships, and increase global connections and networks

**Fig. 5:**
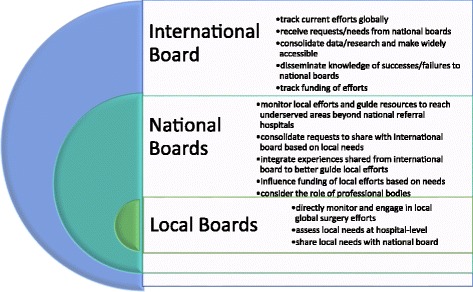
Roles of a Single Multi-Level Global Surgery Administrative Structure. Local, national, and international boards working in conjunction as a single multi-level administrative structure can unify ISTCs and other sustainable global surgery efforts. Working at a grassroots level, local global surgery boards can directly monitor and engage in local global surgery efforts, assess local needs, and share these needs with the relevant national global surgery board. These national boards can then monitor local efforts and guide resources and funding to underserved areas, while consolidating requests to share with the international board. The international board tracks global surgery efforts, receives requests/needs from the national level, consolidates data/research, tracks funding, and disseminates data and knowledge of successes/failures down to national boards, who can then share this global information to those working on the local level

Lack of organization and a systematized process for involvement in ISTCs is a major barrier to their success. This section describes suggestions/models that can be implemented to better organize ISTCs, based on participants’ interviews. Figures 1, 2, 3, 4 and 5 summarize and consolidate the points below in a framework for a unified approach to ISTCs and sustainable global surgery efforts.

### A website for organizing and consolidating information

A centrally accessible website with comprehensive information on ISTCs could list surgical healthcare providers worldwide who are engaged in global surgery work, along with their specialty/subspecialty, where they are from, where they are going, and other pertinent information. This could allow surgical healthcare providers to easily find opportunities for involvement. This could also provide a way for those involved to contact each other and to learn from each other’s work, while also creating a platform for collaboration and new initiatives. See Fig. 1.

Although this is a difficult task, it may be more easily facilitated by the involvement of national surgical professional bodies. Professional bodies are well-respected by surgical healthcare providers and have a sense of academic activities happening within a country or region, so they have the ability and capacity to call for and organize information on global surgery efforts. As these bodies are not affiliated with any particular institution, they serve as an impartial resource for centralizing data and information, so surgical healthcare providers may be more willing to share information about their global surgery work.

### A cyclic process of action and reflection to facilitate communication

More time should be spent reflecting on work related to ISTCs. After any on-the-ground experiences within an ISTC, there should be an open meeting to reflect on what worked well and what did not, as well as other lessons learned. These lessons from previous experiences can be applied to future work to improve efficacy. Integral to this process is the involvement of local stakeholders, such as surgeons, nurses, and other healthcare providers, who can provide constructive feedback on the elements of the partnership that are going well and those that are not. For example, Human Resources for Health in Rwanda has gained valuable insight from introspective reflection of their program’s growth [27]. Additionally, many participants had also reported that people who would consider becoming involved in ISTCs remain unaware of their options for participation. Reflection meetings could not only improve collaborations, but could increase the number of participants, which could help advance global surgery efforts [28].

On a much larger scale, there should be involvement of and alignment with in-country stakeholders such as the Ministry of Health, Ministry of Finance, etc. who can lend support to ISTCs, direct resources to the areas of most need, and guide where efforts are implemented. This could potentially take form as an expansive country-wide conference that includes all HIC teams involved in the region as well as all local stakeholders to reflect on the coordination of efforts and how best to address the country’s needs. More recently, conferences such as these have been held in countries such as Zambia and India, leading to collective involvement of surgical healthcare providers and national stakeholders in discussing each country’s surgical needs and ways to address them [29]. These conferences are often organized and hosted through the collective efforts of both LMIC and HIC stakeholders, including surgical healthcare providers and the Ministry of Health, as part of the implementation of national surgical, obstetric, and anesthesia plans [29].

Ultimately, any form of reflection on ISTCs and their progress can better facilitate communication amongst involved parties, as well as facilitate coordination and collaboration amongst current efforts. By involving local surgical healthcare providers and national stakeholders, information from the local level can drive learning while being integrated at the national level to reflect on the progress of the country overall. This can help direct the work of ISTCs and better unify efforts. See Fig. 2.

### A scholarly approach

Participants called for a scholarly approach to ISTCs. Acknowledging the academic relevance of global surgery can lead to more significant improvements through increased participation in education, research, and service related to global surgery. Academics specifically called for interdisciplinary collaboration in global surgery efforts, to include fields such as health education, sociology, economics, and anthropology in order to make sure ISTCs are yielding the best results via the best approaches [30].

Currently, there is insufficient research in the field of global surgery, especially regarding the effectiveness of capacity-building partnerships [31]. Needs assessments should be developed by those in LMICs, so that ISTCs can be directed to address LMICs’ specific needs. Attention should be given to cultural humility of global surgery interventions, which is important in ensuring that the interventions align with the goals of LMICs. Assessments of education methodology and technology are important in addressing how best to teach surgery in resource-limited settings. Equally relevant is the need for longitudinal outcomes research that assesses whether ISTCs are producing successful outcomes, including increased number of surgical healthcare providers in LMICs, greater number of surgeries, and improved patient outcomes. There is also a need for a standardized system of metrics to evaluate these outcomes in order to inform policy makers [32]. Increased research output in regards to global surgery is a feasible step towards improving efforts such as ISTCs and can simultaneously serve to increase the presence of global surgery in the academic arena.

Program evaluation should be a systematic part of ISTCs, and recommendations from both local and visiting teams should be taken into account during this evaluation. Reports should outline what the needs and goals were, whether those needs were addressed, how the goals were met, what was successful, what was unsuccessful, and what the follow-up plans are. To facilitate collaboration, these reports should be publicly shared so that others can learn from the findings. These types of evaluations can help prevent duplication of unsuccessful models and can instead promote improvement and effectiveness. Additionally, establishing an equal and bidirectional partnership is important to achieving success, which is why evaluation from both sides of the partnership is essential [33]. Program evaluation in a bidirectional manner is already being done by some who are involved in ISTCs, but it should continue and become a more regular part of global surgery work such that these efforts can further improve. See Fig. 3.

### Technology for Continued Long-Distance Collaboration

Global communication technology can be integrated into ISTCs in order to provide year-round education over long distances. Technology allows for a continued presence of surgical healthcare providers from HICs in LMICs, which encourages collaboration and formalizes partnerships, while accelerating and continuing learning [34, 35].

Skype can be used for telementoring and patient consults, so that surgeons in LMICs can work with those in developed countries to discuss cases, share scans, and receive clinical advice. Google Glass, Wi-Fi enabled video cameras, and YouTube can be, and in many cases have already been, used to broadcast patient rounds and surgeries to students both in HICs and LMICs. Showing operations can help with learning new procedures, solidifying knowledge of previously learned procedures, and gaining exposure to more cases. Some surgeons are also using telesimulation to teach surgical skills to those in LMICs [36, 37]. Participants pointed out that technology is being implemented by many who are engaged in global surgery work and has been a feasible addition to ISTCs. Although patient privacy is a concern, measures can be taken to ensure that privacy laws are followed and patient consent is obtained.

The Internet can be used to create online teaching modules in surgical specialty education to supplement the hands-on education of surgical healthcare providers in LMICs [38]. It can also be used to disseminate knowledge via shared textbooks, journal articles, and other less accessible materials [38]. Online curriculum modules and educational materials have been successful in supplementing and standardizing thoracic surgery in the U.S., and this model could potentially supplement education in LMICs [39]. This increased access to knowledge is invaluable in improving surigcal care and facilitating the education of more surgical healthcare providers worldwide. See Fig. 4.

Technology, however, should be used with caution, as mentioned by the study participants. It should not be used as a substitute for personal interaction and on-the-ground work in LMICs, but rather as a supplement to maintain a partnership and reinforce learning [34]. Face-to-face interaction during ISTCs allows for surgical procedural education that is adapted to local realities, as well as for the development of close friendships and stronger partnerships.

### A systematic approach of expansion and consolidation

ISTCs often begin with individual efforts, and as mentioned by participants, this can be discouraging since there is no systematic method for involvement and implementation. Individual ISTCs are often done in many different ways, but a more structured approach is needed to globally coordinate efforts [18–21]. This coordination could happen at the national level, through discussions with the Ministry of Health, Ministry of Finance, and healthcare representatives from different regions in the country. This could also involve professional bodies who are monitoring surgical activities in the country or region and can convey information to national governmental bodies. A brief overview of a proposed systematic process is outlined below and in Fig. 2: expansion and consolidation phases.

In order for ISTCs to have a greater impact globally, more ISTCs need to be formed, which is the goal of expansion phase. This includes the process of reaching out to new potential sites and initiating contact. The consolidation phase, which supports sustainability, includes maintaining partnerships through a continued presence. This is the phase most ISTCs are currently in. Sites should be revisited, so that there is assurance that new techniques, surgical education programs, and other interventions have been sustained, and so that relationships are strengthened. Vital to consolidation phase and the sustainability of ISTCs is the process of training the trainers – involving local stakeholders to create future plans for expansion of ISTCs to other institutions in the country as well as to more rural regions. This increases capacity for “south-south” partnerships between LMICs as well. See Fig. 2.

Once an ISTC has met these goals for a particular site, expansion phase can be repeated. However, repeating expansion phase does not imply termination of relationships that were maintained in consolidation phase, but instead focuses on a comprehensive picture of how more relationships can be formed. These cycles of expansion and consolidation could allow ISTCs to both proliferate in number and be sustainable with long-term successful outcomes. This is essential so that efforts are not only reaching tertiary hospitals, but are also building surgical capacity in rural hospitals and clinics [40].

As mentioned, there are significant advantages to maintaining partnerships long-term, especially given their bidirectional nature. While helping build surgical capacity in LMICs, surgeons from HICs learn how to navigate complex surgical cases in low-resource settings and how to effectively develop training programs. Other benefits from involvement in ISTCs for surgical healthcare providers from HICs include becoming well-rounded and more adaptable in surgery, improving educational skills, and developing teamwork and leadership skills that are beneficial in surgical programs at their home institutions. Overall, continuing a bidirectional exchange of knowledge in ISTCs is important to increasing collaboration and unity in such efforts by equally engaging both sides of the partnership and by maintaining relationships long-term.

This pattern of expansion and consolidation may be more difficult to implement, as it first requires a stronger foundation and systematic process for ISTCs, which may take several years. As more is learned from the implementation and continuation of current ISTCs, this process can become more streamlined.

### A single multi-level administrative body and organization

Participants mentioned concern that organizing ISTCs could lead to bureaucracy– something that surgical healthcare providers often encounter at their own institutions. However, an administrative body to keep track of current efforts, to receive requests/needs, to receive offers to help, to track funding, to collect data/research, and to disseminate results could better unify, systematize, and improve the operation of ISTCs.

A possible model for organization is a three-tiered system: local, national, and international. At each level, there could be a “Board of Global Surgery,” with the local boards feeding information to the national and international boards. This bottom-up approach could help with the dissemination of information regarding ISTCs globally, without restricting individual initiatives. These boards could include groups of individuals that could function to provide organizational support to ISTCs worldwide. On a national/regional scale, there are professional bodies in place that direct, monitor, and evaluate surgical services in many countries around the world, including LMICs. Professional bodies could potentially play a significant role in organizing and unifying global surgery efforts on a national level, and can then feed information to a larger international body that coordinates ISTCs worldwide. On an international scale, a board could monitor and organize these national efforts and serve as an international data collection/research hub for global surgery work [19]. See Fig. 5.

There are, however, some challenges to creating this level of organization. As participants mentioned, some are resistant to centralized organization due to concerns over increased bureaucracy and loss of autonomy in global surgery work. Additionally, it is difficult to determine whether new organizational bodies are needed or whether organization should occur through already existing groups. Although developing this type of organizational structure might be difficult, it is necessary to begin considering fluid models that could ease the process of involvement in ISTCs. Those who are involved in global surgery efforts should work together and expand networks in a unified way [41].

## Conclusion

ISTCs are a long-term sustainable intervention involving partnerships that are geared towards building surgical capacity in LMICs. However, currently, the approach of HICs to these collaborations is fragmented. This study suggests that in order to organize ISTCs, those involved should consolidate efforts, collaborate between groups involved in ISTCs, establish a system of support, take on a scholarly approach, and integrate technology into ISTCs. This study also introduces the possibility of systematizing ISTCs through action and reflection, expansion and consolidation, and a single multi-level administrative organization.

The overarching goal of ISTCs is to improve access to surgical care and to reduce the morbidity and mortality from surgical conditions in LMICs. Better coordination of efforts in specific ways can improve the number, scope, and sustainability of ISTCs in an attempt to achieve that goal. Future studies could analyze specific collaborations and assess their progress in regards to the implementation of the above recommendations and framework. They could also address the practicality of these interventions and the implications of their implementation. A similar study should be done to address the LMIC perspective of ISTCs and other sustainable global surgery efforts such that the results from both studies can lead to overall improvement and better coordination of ISTCs worldwide.

## Additional file

Additional file 1: Complete Interview Guide Used for Data Collection. (DOCX 91 kb)

## Abbreviations

HICs: High-income countries
ISTCs: International surgical teaching collaborations
LMICs: Low- and middle-income countries
