# Supplementary material for: Unifying a fragmented effort: a qualitative framework for improving international surgical teaching collaborations
Source: Global Health. 2017 Sep 7;13:70. doi: 10.1186/s12992-017-0296-7 (PMC5588718; doi:10.1186/s12992-017-0296-7)
Supplement: Additional file 1: — Complete Interview Guide Used for Data Collection. (DOCX 91 kb) [file 12992_2017_296_MOESM1_ESM.docx]

1. Please state your age and where you are from.
2. Gender:
3. What degrees have you earned?
4. What country do you live in?
5. What is your specialty and/or subspecialty?
6. How long have you been practicing in your specialty?
7. Do you have a current research focus/interest? If so, what is it?
8. What do you know about the global need for surgery?
9. What do you think would be most effective in addressing this need?
10. Please describe your involvement in global surgery work, including teaching collaborations.
11. When did you begin your involvement and why?
12. Do you think the current approach to international surgical teaching collaborations is fragmented?
13. If so, why do you think this is the case?
14. Do you think international surgical teaching collaborations should be more unified?
15. If so, what interventions do you think will better unify these efforts?
16. How can we improve international surgical teaching collaborations?
17. What do we need to know about international surgical teaching collaborations in order to move forward with our efforts?
18. What additional research is needed?
19. Do you see technology fitting into international surgical teaching collaborations? If so, how?
20. What hesitations do you have, if any, when using technology as a part of these collaborations?
21. What other ideas do you have on the topic of international surgical teaching collaborations that you would like to share? Any suggestions or ideas that you wish to see implemented?
